# Diseased Conditions—Their Effects upon the Teeth

**Published:** 1874-05

**Authors:** G. V. Black

**Affiliations:** Jacksonville, Ill.


AMERICAN JOURNAL

OF

DENTAL SCIENCE.

Vol. VIII. THIRD SERIES--MAY, 1874. No. 1.

### ARTICLE I.

### *Diseased Conditions—Their Effects Upon the Teeth.*

**Read before the Illinois State Dental Society.**

BY G. V. BLACK, JACKSONVILLE, ILL.

Pathological conditions have not been very closely studied in relation to their effects upon the teeth, and we find our knowledge of them somewhat obscure.

A great many writers have seemingly rested their conclusions upon the merest hypotheses, or upon the views of preceding writers, which we are often unable to trace to any specific observations sufficient to render their opinions authoritative.

What is known of the pathological chemistry of the secretions is much scattered in different works, so that it is difficult to collect them, and often their great variance renders the formation of correct conclusions impossible.

On many points, however, we find among the abler zoochemists who have made pathological investigations, a tolerable agreement, and considerable reliable information as to the changes which take place in the secretions.

A number of theories yet exist as to the cause of caries of the teeth. One point, however, we think, may be considered as established, *i. e.*, that the presence of acids is necessary to its inception and progress. We shall, therefore offer no argument on this point, but, for the most part, confine ourselves to those conditions in which acids are introduced into the oral cavity by way of the secretions, using freely what we have obtained from different authors, with our own observations. The extended citation of authors, however, would render our paper too long. Neither can we do more than give prominent features, with here and there some details.

It is well known that the teeth are harder in old age than in youth; that the texture of the teeth becomes firmer as age advances. It has also been noticed repeatedly that the teeth are liable to considerable fluctuation in this respect; that teeth which had previously been noticed to be very firm in texture are afterward observed to be quite soft.

This softening has been noticed more as occurring in pregnancy than in any other condition, and has been attributed to the withdrawal of the lime salts to build up the bones of the fœtus.

It is very generally supposed that the reason for this drain is to be found in the lack of lime salts taken with the food.

I have frequently noticed that the teeth of consumptive patients become very much softened, which I regard as the direct result of the failure of the nutrient functions. A like result is liable to ensue from any failure of nutrition, such as long-continued fevers, or any great depression of the vital powers. Just what changes take place in the constituents of the dentine in these cases is not definitely ascertained, and this point will doubtless be a very difficult one to settle. We are inclined, however, to the opinion that the phosphate of lime is diminished, while the carbonate may be increased. This theory explains more perfectly the universally admitted fact of their being more rapidly destroyed by caries—the carbonate of lime being more soluble than the phosphate.

Much close observation has convinced us that this softened condition contributes but little to the *inception* of caries, but that it allows decay that has become established to progress with greater speed. For the inception of caries, another derangement seems necessary besides the softened condition of the teeth themselves. *Normal saliva* being *alkaline* in reaction, evidently has the effect of neutralizing any acids that may be brought in contact with the teeth. It therefore follows that acids, to effect the solution of the tooth substance, must be in some manner protected from alkaline saliva. This may possibly happen in some positions between the teeth, but such an occurrence seems hardly probable. Neither does it seem probable that the food lodging between the teeth, and decaying, would, while bathed in an alkaline fluid, be able to produce an acid which would affect the solution of the tooth substance. If, however, in connection with such lodgment, an irritation of the gum be produced, and in consequence an acid mucus be poured out into, and around, the decaying food, we have more reason to suppose that an intensely acid condition may arise, and caries be established. If, however, the entire fluids of the mouth be acid in their reaction, as the result of disease or constitutional anomaly, then we can understand how an intensely acid condition may arise in secluded positions about the teeth which favor the lodgment of food, or of the saliva itself—for this substance is liable to speedy decomposition. In such a case we have no neutralizing element, and no check, either as to acid fermentation or its action on the tooth substance, and therefore caries is readily established. When a breach is made in the substance of the tooth, and something of a cavity is formed, presenting only a small opening, and in consequence an almost statical condition of the contents is maintained, it is plain that the very small addition of saliva will only serve as food for acid fermentation, and the caries will continue to progress, although the saliva may have returned to its normal condition of alkalinity. If, however, the acids have only softened a portion of the substance, and no cavity be formed, or even if the cavity formed have a broad opening, admitting fluids freely, then it usually occurs that the action ceases on the return of the normal quality of the secretions; and the part affected changes to a dark color, forming the spots and so-called "black decays" so often seen in teeth, and so remains, until a renewal of acidity forces the action deeper into the substance of the organ.

If normal saliva is alkaline in reaction, then acidity of this fluid is a diseased condition; and if what we have just said be in anywise true, it is a diseased condition of the utmost importance to the dental surgeon—one which he should labor to understand, and, if possible, to correct.

That the healthy saliva is alkaline, is the opinion of all the great experimenters in zoo-chemistry, among whom we might mention Liebig, Lehmann, Wright, Jacubowitsch, and various others. For five years we have been constantly in the habit of testing the saliva of our patients, and are fully convinced that the views of these men are correct, as applied to our own people. We are not unmindful of the fact that a great many tests have been made, and some reports published, that seem to show that acid saliva was the prevailing condition, to which there was very few exceptions; and for this reason we have been very cautious in our observations, and somewhat slow to publish results.

In making the seemingly simple test of the saliva, very much care is necessary—much more, indeed, than most persons would be likely to admit. In the first place, it is necessary that the test paper should be just right. The color should be uniform throughout, and when dipped into a known neutral fluid, should remain uniform. I have frequently purchased litmus paper that would, when dipped into water, show red spots, or streaks, here and there, over its surface. Such paper will certainly lead to error, and should, therefore, be discarded. The greatest care should be taken to prevent injuring the paper in handling, for it is known that the exhalations from the skin are acid in reaction, and may adhere to the paper in so dry a form as not to color it, but do so quite decidedly upon dipping it into water.

In testing the saliva, any point in the mouth may be selected where the fluid seems most abundant, care being taken not to touch the lips or other parts not thoroughly moistened, otherwise we will be liable to touch mucus that is unmixed with the other secretions, which will often show an acid reaction, when the mixed fluid would be neutral or alkaline. (When we use the term saliva without qualification, we always refer to the mixed fluids of the mouth.) If, when the paper is withdrawn, it shows changes in streaks or spots, it should be thrown aside, and a new test made. There seem to be two reasons for this streaking of the paper, aside from the faulty condition of the paper itself. One is, that the litmus is very readily washed out of the paper by the saliva, and may run in streaks, some parts appearing light and others dark, thereby interfering with the proper reading of the test; or the color may be washed from the paper in such a manner, that the whole surface may appear of a lighter shade, which may also lead to error.

The second cause is from mucus that is unmixed with the saliva, showing a different reaction from the mixed fluid. In ropy conditions of the mucus this is very liable to occur, for the reason that it does not seem to mix with, or dissolve very readily, in the saliva. This feature of ropy mucus renders it comparatively easy to determine its reaction. There is also a difference in the color of the paper when wet and when dry. The best way I know of to learn to read the tests correctly, is to take a vessel of pure water, another with a drop of acid added, and another with a drop of ammonia. Then dip a slip of each of the colors of litmus into the pure water, and lay them on a porcelain slab or a clean white napkin. Dip another into the acid water, and another into the alkaline water, lay them by the first, and note closely the changes. Then add another drop of acid, and one of ammonia, and repeat the operation; and so on until very decided changes are produced. This should be practiced a great deal by any one who wishes to make close and accurate observations by the use of test paper.

In my tests I have, perhaps, found a small majority of the persons under twenty years of age, who present themselves for dental operations, have exhibited an acid saliva, while those thirty years of age and upwards have shown a considerable preponderance of alkaline saliva. Our examinations of persons outside of our operating room show plainly that our patients present a far larger per cent. of acid saliva than the community in general.

A s ngle examination is in no case a sufficient basis upon which to found an opinion as to the habitual reaction of the buccal fluids, for there are frequently presented considerable fluctuations. Saliva which is habitually acid may become temporarily alkaline, from changes in the food eaten; or that which is habitually alkaline may become temporarily acid, so that frequent examinations are necessary to establish the habitual condition. The saliva also presents a greater degree of alkalinity during digestion than after digestion has ceased, and the stomach is empty.

During my examinations, periods have occurred during which a large majority of those examined presented an acid saliva; other periods have occurred during which the opposite was true. Certain families have presented an acid saliva on all occasions, while individuals have presented an alkaline saliva at every examination.

The greatest proportion of acid saliva has been presented in the time of the prevalence of intermittent fevers, when for one month it amounted to 0.80. The smallest proportion of acid saliva was found during the month of February of this year, when it amounted to 0.23. During this month there was but little sickness, and the few cases were mostly thoracic inflammation.

We have examined the saliva of many persons who had never had a decayed tooth, and of those in whose teeth decay had evidently ceased, leaving a portion of the teeth in a condition of usefulness. Such persons have almost uniformly presented an alkaline saliva, and those whom we have frequently examined have all shown an habitual alkaline condition.

In cases where decay was evidently progressing rapidly, and was of light color, we have uniformly found the saliva habitually acid in reaction. In those cases where decay is making apparently rather slow progress, and inclined to be of a dark color about the margins and outer strata of the softened portions, we have usually found the saliva nearly neutral, but vascillating between the alkaline and acid reaction. In cases where the decays all present a black color, and this color extends into the still hard dentine, in those cavities with broad openings, we have very generally found the saliva alkaline. In this last condition, those cavities which have very small openings closed by adjoining teeth, often present a lighter color in their interior parts, and are evidently progressing ; but those that are black throughout we believe to have become stationary, and will make no progress while the alkaline condition of the saliva remains.

It is not unusual, by any means, to find a number of very dark decays in the mouths of persons somewhat advanced in life, that have made no progress for many years. Then there must be some substantial reason for this cessation of decay. The cause of cessation cannot be found in any change in the structure of the dentine, for it is certainly undeniable that all classes of teeth, the hardest and softest, are liable to decay; not only this, but we have frequently observed it in teeth that had lost their pulps when the decay was progressing—which precludes all possibility of changes in their dentine for the better ; then the changes which have suspended the action of decay must be found in the buccal fluids.

The *mucus* is not unfrequently found deranged, while the other secretions present a normal condition. These derangements arise, for the most part, from diseased conditions of the membrane secreting it, and have, we think, been more accurately described by Tomes and Wedl than any other writers. Irritations of the mucus membrane produce acidity of the mucus. Thus, if food lodge between the teeth in such a shape that an irritation of the gum be produced, an acid condition will arise about it on account of the irritated gum throwing out an acid secretion, but if decided inflammation be produced, a serum will be exuded, which is nearly neutral or alkaline in reaction; while, if the inflammation pass to suppuration, and pus be formed, it is always alkaline in reaction. It is therefore, slight irritations of the mucus membrane which induce decay, and not active inflammations.

There is a very ropy tenacious mucus met with in some individuals, which often presents a markedly acid reaction; and is evidently the exciting cause of decay in their teeth. This mucus is occasionally secreted by the entire mucus membrane of the mouth, but we most frequently find it coming from some particular part, as from the gums or the surface covered by a plate for artificial teeth, or other parts. The membrane of the part is usually slightly thickened, and presents a somewhat more reddish color; but frequently by no means distinct; so that if the whole membrane be involved, it could not be detected by these features. It frequently happens that this mucus dissolves very slowly in the other secretions, and is found coating the gums, giving them a peculiar slippery feeling to the fingers when applied to them. This condition is most frequently found confined to the gums on the facial side, and gives rise to decay about the necks of the teeth. We have seen a few cases where it extended entirely around both arches, but it is usually confined to particular portions.

The mucus in usually less strongly alkaline than the saliva indeed a good many writers have given slight acidity as its normal condition; but this we think a wrong conclusion. It is, however, often found acid, when no disease of the mucus membrane is apparent. This may happen when the secretion from the salivary gland is normal, and may be detected by pressing the litmus to the gums when but slightly moistened. We generally find the mucus acid, in connection with acid saliva, and it is frequently difficult to determine without examining the secretions separately, to which secretion the acidity of the mixed fluid belongs. We have occasionally found the mucus alkaline, however, when the other secretions were acid.

A variety of forms of disease are accompanied by an acid condition of the buccal fluids. For information as to these, we are for the most part dependent upon the researches of Lehmann, Wright, Jacubowitsch and others.

I have made many observations myself, with generally the same results. It must be remembered, however, that acid saliva is so common in this country that an observation during sickness is not of much value, unless the previous, or habitual condition is known; a fact which renders many of my observations valueless.

In fever and ague, and those kindred affections known as resulting from miasma, the saliva seems to be uniformly acid in reaction, often markedly so. My own observations seem to show that the saliva may become acid from this cause, while the general system is apparently unaffected; for I have certainly found a much larger proportion of acid saliva when they abounded, than at other times. This class of disease is also accompanied by a species of hyperæsthesia, which is a source of much trouble to the dental operator. The teeth are apt to be unusually sensitive, and the patient so nervous as not to be able to bear the excavation of cavities with any degree of fortitude. It is unusually difficult to make a perfect operation, while the acid saliva searches out every fault to undermine and destroy the work we have accomplished. Teeth are liable to ache, and give trouble, that would not do so in better conditions of the system; and pulps capped are more liable to die.

Continued fevers are usually accompanied by an acid condition of the saliva. The mucus also is usually quite markedly acid. As the fever progresses, the secretion of saliva from the salivary glands, is partially, sometimes wholly suspended, and the acid mucus dries upon the tongue and teeth, forming sordes, with which the teeth are often coated for a considerable time.

This seems sufficient cause for the increased amount of decay so often observed following continued fevers; especially in young persons, and which is so often attributed to the effects of medicines.

Irritations and inflammations in the course of the alimentary canal are accompanied by an acid saliva. Although their course is usually short, their occurrence is quite frequent, and I have but little doubt that they have much influence in the inception of decay in the teeth of young persons.

Diabetes is always accompanied by an acid saliva, which is usually very marked. Leber and Rottenstein, in their recent work, contend that the acid reaction in this case results from the acetous fermentation in the mouth, of the sugar contained in the saliva in this disease. But Lehmann has drawn the saliva directly from the gland into alcohol, so that such fermentation could not take place, and yet he found it acid.

In irritations and inflammations of the uterus, the saliva is acid, sometimes markedly so. It is also very generally acid during pregnancy, frequently continuing during lactation. We have but little doubt but that this is the true cause of the increased decay of the teeth, which is so marked in pregnant and nursing women. The softened condition of the dentine renders the destructive process more rapid than it would otherwise be.

In dyspepsia there is much disagreement among authors as to the conditions of the secretions of the mouth. We think, however, that two forms of dyspepsia have been confounded, one presenting acidity, and the other the opposite condition of both the oral and gastric fluids; certainly we have found very opposite results in our own observations, both as to the condition of the saliva, and the effects upon the teeth; some cases showing very acid saliva, and the teeth decaying rapidly, while others show an alkaline saliva, and no decay is manifested.

Consumption is usually accompanied by acid saliva.

In thoracic inflammation there seems to be an increased alkalinity of the buccal fluids. Lehmann has always found them alkaline, while Wright and Jacubowitsch have sometimes found them acid.

In inflammation of the brain and nervous system, the saliva is alkaline.

So far there is a tolerable agreement among chemists who have made pathological investigations; but in most other diseases, the disagreement is so great that no definite conclusions can be formed.

The causes of habitual acidity of the saliva are not as yet understood. It seems to be in some degree an accompaniment of civilization; yet it cannot be a necessary result of our mode of life, as it is by no means universal. Cattle and horses, stall-fed, in close stables, often present it, while those that feed naturally do not. From the fact that we often find it to exist in certain families, every member presenting it, while others present it only occasionally, we are of the opinion that it is an hereditary anomaly, or disease, and that this is the true source of the hereditary nature of caries of the teeth. In all cases where I have found persons of forty or forty-five years of age, with a continuance of habitual acidity of the buccal fluids, without irritations of the mucus membranes, or other visible signs of disease to produce it, I have found their children presented it also.

Habitual acidity is also acquired in some manner where there is no such transmission, and may be of long duration. Many of the cases are, however, temporary in their character, lasting for a few months, weeks, or even only for a few days, and there are, perhaps, very few persons who do not, at some time, present it. The greatest fluctuations are in youth, while in middle and advanced age examinations, from time to time, have shown but very little change.

We cannot now point out any particular quality of food that is certainly known to produce acid saliva without other marks of disease. Some few facts, however, seem to point to common salt as one of its causes. It is understood by our noblest zoo-chemists that a portion of the metallic chlorides are decomposed in the system, in some manner not yet understood. It is supposed that hydrochloric acid is one of the results, as this acid is usually formed upon the decomposition of the metallic chlorides in the presence of moisture. It is in this way that Lehmann and others account for the frequent substitution of hydrochloric acid for the lactic acid of the gastric juice.

A considerable portion of the chloride of sodium taken with the food fails to re-appear in the excretions, and is therefore decomposed. Dalton places the amount thus lost at one-fifth. We have seen persons who, we have no doubt, would consume, in various ways, as much as an ounce of salt per day. A calculation shows that the decomposition of one-fifth of this would produce sixty-grains, by weight, of gaseous hydrochloric acid.

With these facts before us, we have been induced to try some experiments with common salt. Our own saliva being habitually alkaline, and not having shown an acid reaction for several weeks, we commenced taking an additional amount of salt with our food, amounting to about one-third of an ounce per day. For the first few days the saliva showed a somewhat stronger alkaline reaction. On the evening of the third day it was about neutral, and on the fourth day it was distinctly acid. The salt was not taken after the fifth day, but the acid reaction remained for six days. Another experiment was tried with practically the same result. While these theories and experiments are not sufficient to prove that the free use of common salt is one of the causes of acid saliva, they certainly give us good reason to suspect it, and it can be determined by direct and persistent experiment.

Acidity of the saliva is often accompanied by a scarcity of the secretion, and is probably, in some measure, due to a debilitated condition of the glands; yet in these cases any unusual excitant is apt to induce a superabundant secretion, which, in dental operations, is frequently enormous. In these cases we have generally found the mouth unusually dry, except when there was some special excitement of the glands, and the presence of food fails to excite the usual flow. Even where persons do not complain of dryness of the mouth, an examination reveals but little saliva, so that it often requires some little time to properly moisten a piece of litmus. Such persons are apt to suffer more than usual from thirst. This condition may be in some degree due to the habit of taking liquids while eating, thereby habitually relieving the glands of their natural stimulus of the presence of dry food in the mouth.

The treatment of acid conditions should not be passed without consideration, although nothing very satisfactory can as yet be adduced. Some agents have been found, however, that seem to give some promise of usefulness, although they have not as yet had sufficient trial for us to speak so decidedly of their merits as we could wish.

Acids, administered as medicines or taken with the food, produce increased alkalinity and increased flow of the saliva. Wright found that acids introduced into the stomach of a dog through a gastric fistula also produced this result promptly, and was led to the belief that one office of the saliva was to regulate the amount of acid in the gastric secretions. Experiments with alkalies, however, failed to produce acidity of the saliva; consequently this hypothesis was not maintained.

We have repeatedly found the saliva alkaline a short time after eating oranges and lemons, when it was known to have been habitually acid. But we have not made any very extended trial of the use of acids for the purpose of removing habitual acidity of the oral secretions. It seems, from what we have been able to learn of their action, that their continued use, in large amounts, finally produce opposite effects, in diminished flow of saliva, thirst, dryness of the mouth, and finally acidity of the secretions. Not unfrequently, however, they produce salivation.

There cannot be much doubt but that the properly guarded use of acids in continued fevers is beneficial to the teeth, as they tend to induce a flow of alkaline saliva, by which sordes is neutralized and washed away.

The remedies we have tried most are black pepper, mustard, and armoracia, or horseradish. Either of these, when partaken of pretty freely as condiments, seem to change the condition of the saliva, when it is acid, quite promptly. Of the three, the armoracia seems to have the most decided action. We know of no valid objection to the use of either of them. They can therefore be alternated, as the tastes of the patient may dictate. I have prescribed them to be taken as freely with the food as the tastes would allow ; while salt is avoided as much as possible.

One lady who had, in her two former pregnancies, presented a very acid condition of the secretions, and in each case quite a number of new decays, followed this case with a third. Her saliva was alkaline at the times when I saw her, and no decay made its appearance.

In another case, a lady about twenty-two, unmarried, who suffers much from disease of the uterus, and presents markedly acid saliva and much decay of the teeth, has succeeded in preserving alkaline condition by this course, except at times, when, through sympathy, the stomach is too much deranged to take food. She has pursued this course for about ten weeks.

In most of the cases in which this treatment has been tried, the results have so far been favorable; yet we cannot say whether it will succeed in removing the constitutional anomaly of acidity, so that the remedy can be discontinued, and the alkalinity continue to prevail. If these, or other remedies yet to be found, will bring about this result, we believe caries of the teeth can be controlled with comparative ease. It will, of course, take considerable time to demonstrate satisfactorily the usefulness of any such mode of treatment.

Sialagogues, so far as we have tried them, tend to promote a normal condition of the saliva. The same may be said of the tonic stimulants. Indeed, anything which tends to the promotion of digestion seems to induce alkalinity of the saliva. It seems probable, also, that some of the diuretics may be found useful. Anything which rouses the salivary glands to action seems to induce the elaboration of a normal secretion. Sudden shock, or the endurance of severe pain, as in operations in the mouth, usually induce a temporary change.

We have often noticed that the saliva would be acid before an operation, and alkaline afterward. How much of this may be due to the greater flow of saliva overcoming an acid condition of the mucus, we have not always been able to determine. In many cases, however, where we have examined the parotid saliva separately, we have found a change in that secretion.

The *treatment of acid conditions of the mucus* arising from local irritations is sufficiently clear. All causes of irritation should, of course, be removed, and a healthy condition restored by the usual means. All overlaps of gold or other fillings about the gingival margin of the gum should be scrupulously removed, and the surface made absolutely smooth with that of the surface of the neck of the tooth, so that it cannot act as a mechanical irritant, always remembering that slight irritations are more likely to induce decay than decided inflammations, while the patient is much less likely to notice them.

The *cause of ropy, acid mucus* is not understood. It seems to arise spontaneously, run its course, and cease, after a longer or shorter period. If it continues long the teeth suffer greatly. Those decays caused by it on the facial surfaces of the teeth are very difficult to arrest by filling, as the decay very often reappears by the side of the plug. Indeed, our experience has been that the whole facial surface of the tooth bordering the gum will inevitably be destroyed if this condition remains constant for a considerable period. It is probably the most difficult form of decay to manage known to the dentist.

We have seen this condition arise and cut grooves across eight or ten teeth, disappear, and the decays turn dark—giving evidence of having become stationary within a few months; and again we have seen it continue for years. Again, it disappears only to return again after a few months, to again disappear and again return. Under my observation, it has but rarely remained constant for a very long period.

The thickened and slightly ingested condition of the membrane indicates the use of astringments in its treatment. We have, however, relied more upon friction, in the cases which have come under our care, and have found it very effective in removing it. We direct that the part be thoroughly and strongly rubbed with a quick motion, once or twice per day, either with a rough cloth or a pretty stiff brush.

